# Principles for the production and dissemination of recruitment material for three clinical trials of an online intervention

**DOI:** 10.1186/s13063-021-05412-4

**Published:** 2021-07-09

**Authors:** Stefan Rennick-Egglestone

**Affiliations:** School of Health Sciences, Institute of Mental Health, University of Nottingham, Nottingham, UK

**Keywords:** Participant recruitment, Online recruitment, Social media, Health research

## Abstract

Some health research studies recruit participants through electronic mechanisms such as the placement of messages on social media platforms. This raises questions for ethics committee oversight, since effective social media campaigns might involve the production and dissemination of hundreds of contemporaneous messages. For the Narrative Experiences Online (NEON) study, we have developed nine principles to control the production and dissemination of promotional material. These have been approved by an ethics committee and enable the audit of our recruitment work. We propose that the drafting for approval of recruitment principles by health research studies may, in many cases, strike an appropriate balance between enabling ethical oversight of online recruitment work and the potential burden of message review.

**Supplementary Information:**

The online version contains supplementary material available at 10.1186/s13063-021-05412-4.

## Main text

Some health research studies recruit participants by disseminating recruitment material through electronic mechanisms. This material might include short messages disseminated in invitation emails or on social media platforms such as Facebook [1, 2]. It might also include more detailed study information distributed through websites such as clinicaltrials.gov [3]. In some cases, online dissemination of recruitment material can enable rapid recruitment of participants [4] and might also allow access to groups who are otherwise hard to reach [5]. It does of course risk excluding people who have difficulty accessing the Internet, perhaps for economic, cultural, social or personal reasons. This phenomenon has become known as “digital exclusion” [6].

Online dissemination of recruitment material raises some specific ethical issues. For example, interview evidence suggests that researchers who “lurk” on online health support forums to post recruitment messages can harm their capacity to act as a safe space for discussion [7, 8]. Whilst social media recruitment campaigns might benefit from the production and dissemination of a large number of messages tailored to the interests of different groups [9], the review of the content of these messages by an ethics committee might be untenable at a scale that enables recruitment success. Although regulatory bodies typically specify clear approval processes for “traditional” recruitment media such as posters, we have found that approval processes can be ambiguous for online recruitment campaigns. This risks an unprincipled variation in the approaches that ethics committees are willing to approve. It also risks uncertainty about the approval status of recruitment messages.

The Narrative Experiences Online (NEON) study is currently (as of March 2020) conducting three clinical trials [10] of the NEON Intervention [11], an online mental health intervention designed to improve quality of life by providing access to a collection of mental health recovery narratives [12]. All procedures for the NEON trials are conducted online, including the collection of consent, baseline and follow-up data [10]. In keeping with these online-only trial procedures, we have recruited at least 75% of our participants through online mechanisms, including through the placement of paid promotion on social media platforms and websites, and the distribution of electronic messages to more than 1000 community groups. All of our messaging has been constructed as part of targeted “campaigns”, such as the “last push campaign” in which we constructed and disseminated messages indicating that limited spaces remained in our trials and the “diversity campaign” in which we worked with community champions to disseminate messages targeted at people who identify with non-majority demographic characteristics. The NEON trials have recruited to time and target. We believe that the incremental effort of posting large numbers of tailored messages has contributed towards this success, and will evaluate this in our process evaluation [10].

To allow for ethical oversight of our recruitment work, we developed 9 principles to control the production and dissemination of recruitment messages. These principles are referenced in our trial protocol [10] and were approved by an ethics committee in advance of our trials opening. They are presented in Table 1.

**Table 1:** Nine principles of recruitment material design selected for the NEON trials

ID	Principle
1	If the communication mechanisms afford it (e.g. on a poster), then promotional material will include the study sponsor logo and name, the study logo and details of the approvals received by the study (e.g. Health Research Authority, name of REC offering favourable opinion, study sponsor).
2	If the communication mechanism does not afford it (e.g. in a tweet with limited characters), then the promotional material will always include a link to a page that provides the same information as in principle 1.
3	Promotional material will clearly indicate that we are looking for participants for a clinical trial (“trial” may be used as an informal synonym of “clinical trial”).
4	Promotional material will clearly indicate that the trial involves receiving recovery stories (the term “recovery story” has been selected as a more accessible synonym than “recovery narrative”).
5	If images of people are included in the promotional material, then these will only be included if appropriate documented consent is in place for this usage, e.g. if the image was specifically captured for inclusion in the promotional material, or if it was licensed from an image library (e.g. a stock image of two people working on a computer).
6	If the communication mechanism affords it (e.g. a poster), then typography and layout will be selected to be dyslexia-friendly and appropriate for people with red-green colour blindness, as this is the most common form of colour blindness.
7	Promotional material will not be placed by the study team into settings where people have a reasonable expectation of privacy (such as Facebook groups closed to public membership). Promotional material may be placed into private settings only by people who have a reasonable, pre-existing right of access to those settings (such as existing members of Facebook groups).
8	Promotional material will not be made available in languages other than English, even on request, as fluency in English is an inclusion criterion for all three trials.
9	All promotional material used by the study will be archived in the TMF, and hence will be open to audit by the study sponsor, so that the study sponsor can confirm that these principles have been applied.

These principles were selected to serve the following purposes: ensuring that potential participants have access to appropriate and coherent trial information (principles 1–4 and 8), avoiding misuse of personal images (principle 5), encouraging inclusive design [13] (principle 6), precluding lurking for the purposes of recruitment (principle 7), and enabling an audit process for messaging (principle 9). These purposes were selected by the NEON research team as being the most important to address for our trials. Our experience of putting these principles into practice is that they have enabled rapid production and dissemination of recruitment messages and also enabled team discussion and knowledge development around ethical recruitment strategies.

We propose that this model—of agreeing specific principles for online recruitment work with ethical oversight bodies—is transferable to other health research studies and that it might be an appropriate solution for efficient use of online recruitment methods, whilst providing guarantees that online recruitment will be conducted ethically.

Whilst some principles might be common to a range of studies, researchers developing study approval applications might need to select a subset of principles that are most relevant to their population and study design. A community effort to identify and disseminate principles for online recruitment might support the production of ethically sound study approval applications. This approach as a whole might support studies in recruiting to time and target.

## Supplementary Information

**Additional file 1.** Recruitment principles for the NEON trials. Document presenting the nine principles controlling the design of recruitment material for the NEON trials.

## Data Availability

Not applicable.

## Abbreviation

NEON: Narrative Experiences Online
